# To transduce a zebra finch: interrogating behavioral mechanisms in a model system for speech

**DOI:** 10.1007/s00359-017-1153-0

**Published:** 2017-03-07

**Authors:** Jonathan B. Heston, Stephanie A. White

**Affiliations:** 1 Interdepartmental Program in Neuroscience, University of California, Los Angeles, Los Angeles, CA 90095 USA; 2 Department of Integrative Biology and Physiology, University of California, Los Angeles, Los Angeles, CA 90095 USA; 3 Present Address: Department of Neurosciences, University of California, San Diego, San Diego, CA 92093 USA

**Keywords:** Birdsong, Medium spiny neuron, Pallidum, Striatum, Virus

## Abstract

The ability to alter neuronal gene expression, either to affect levels of endogenous molecules or to express exogenous ones, is a powerful tool for linking brain and behavior. Scientists continue to finesse genetic manipulation in mice. Yet mice do not exhibit every behavior of interest. For example, *Mus musculus* do not readily imitate sounds, a trait known as vocal learning and a feature of speech. In contrast, thousands of bird species exhibit this ability. The circuits and underlying molecular mechanisms appear similar between disparate avian orders and are shared with humans. An advantage of studying vocal learning birds is that the neurons dedicated to this trait are nested within the surrounding brain regions, providing anatomical targets for relating brain and behavior. In songbirds, these nuclei are known as the song control system. Molecular function can be interrogated in non-traditional model organisms by exploiting the ability of viruses to insert genetic material into neurons to drive expression of experimenter-defined genes. To date, the use of viruses in the song control system is limited. Here, we review prior successes and test additional viruses for their capacity to transduce basal ganglia song control neurons. These findings provide a roadmap for troubleshooting the use of viruses in animal champions of fascinating behaviors—nowhere better featured than at the 12th International Congress!

## Introduction

A powerful tool for testing nervous system function is the experimental manipulation of genes in the brain. Knocking down, knocking out or overexpressing an endogenously expressed gene can allow one to gain insight into its biological role. The ability to overexpress exogenous molecules such as fluorophores or activity-manipulating molecules such as opto- or chemo-genetic ones has been critical in mapping neural circuits and understanding the relationship between neural activity and function. To date, however, the use of such strategies in the avian nervous system has been limited. Yet, the ~9000 extant species of birds display a vast array of fascinating behaviors including: Technological feats such as the ornamented nest building of the satin bowerbird (*Ptilonorhynchus violaceus*) and sophisticated tool making in New Caledonian crows (*Corvus moneduloides*); physical feats such as the prodigious 90,000 km (56,000 mi) migration of the arctic tern (*Sterna paradisaea*) to the lek-based explosive gymnastics of the golden-collared manakin (*Manacus vitellinus*); and the capacity for vocal learning exhibited across three avian orders: Hummingbirds (*Trochiliformes*), parrots (*Psittaciformes*) and songbirds (*Passeriformes*), perhaps most famously exemplified by the lyrebird (*Menura novaehollandiae*). For those species that are amenable to a laboratory environment such as the zebra finch songbird (*Taeniopygia guttata*), the ability to genetically manipulate the circuitry underlying these behaviors offers a functional test of the key physiological specializations.

One avenue of gene manipulation commonly used in rodents is transgenesis. The major hurdle in generating a transgenic bird is the egg which provides a physical barrier against accessing the developing embryo. Several studies have overcome this hurdle by opening the egg, injecting lentivirus to induce transgenesis in the pro-gametic cells of the developing embryo, and then closing the egg and allowing it to hatch, as notably first performed in chickens (*Gallus gallus domesticus*; McGrew et al. 2004). Pioneering work in songbirds by Agate et al. (2009) provided a proof of principle of this strategy to generate zebra finches that overexpress the reporter gene for green fluorescent protein (GFP). More recently, additional transgenic zebra finches have been created to over or under-express the neuronal plasticity-related transcription factor, cyclic AMP response-element binding protein (CREB; Abe et al. 2015), and to model a human neurodegenerative disorder by expressing mutant Huntingtin protein (Liu et al. 2015). This strategy, however, has an almost prohibitively low success rate (1–2%) to make this a viable option for most researchers.

Transgenesis can have other limitations that make it non-ideal even if it had a higher success rate. First, basic transgenesis lacks temporal specificity and thus molecular changes are generally altered throughout the life of the organism, including during development, provided that the manipulation itself is not embryonically lethal. Fortunately, inducible promoters help to address this drawback. Second, it can lack spatial specificity, altering gene levels globally including numerous off target regions and cell types. Cell-type specific promoters help to address this drawback, but may still be ‘leaky’ given that the exact regions of promoter sequence that ensure specificity can extend hundreds of kilobases (kb) upstream of the start codon (e.g. Bruce and Margolis 2002). With regard to vocal learning birds, in the absence of inducible promoters, continuation of transgenic lines can be problematic because any manipulation that leads to song learning errors can lower mating success given that song is a reproductive behavior.

**Fig. 1:**
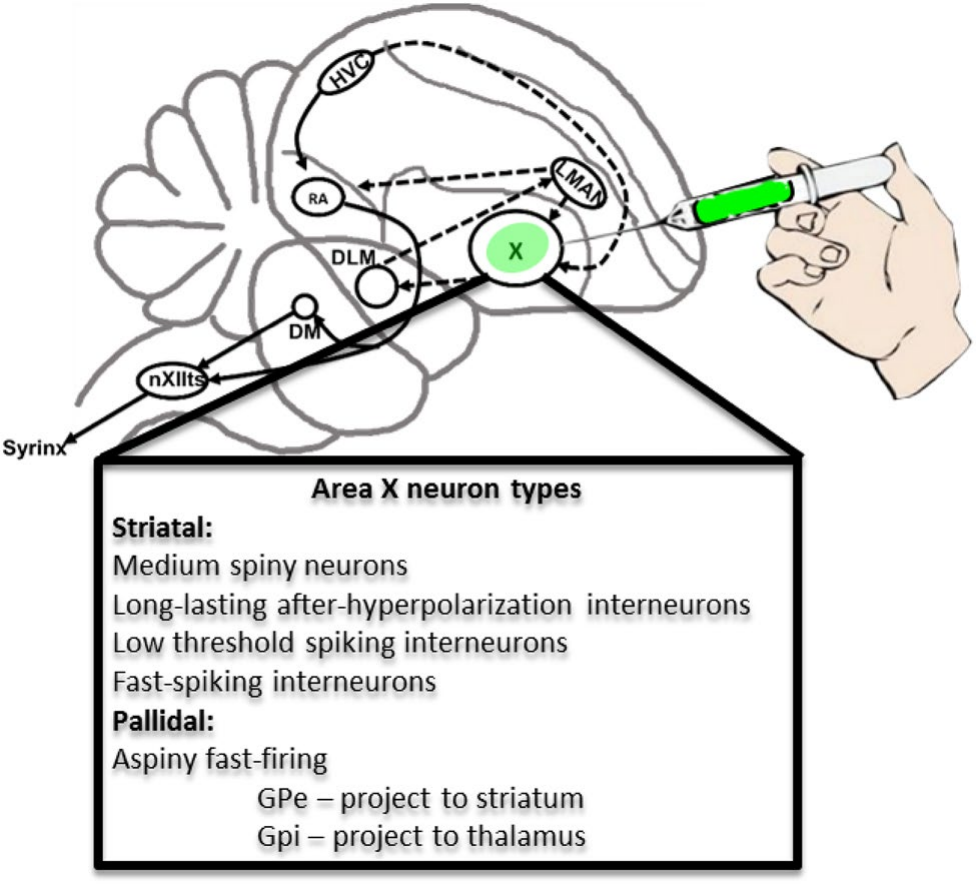
Song control circuitry including neuronal phenotypes of striato-pallidal Area *X*. Sagittal schematic illustrates major components of the song control system. The basal ganglia nucleus dedicated to vocal learning, Area *X*, is shown as the target of stereotaxic injections with viruses that encode fluorescent reporter proteins. Abbreviations are described in the list. Below, the basic neuronal phenotypes that comprise Area *X* are shown (Farries and Perkel 2002; Farries et al. 2005; Goldberg and Fee 2010a, b)

While the technology for producing transgenic birds will undoubtedly improve as it has for rodents, and methods including CRISPR will eventually overcome these hurdles, viral mediated gene transfer currently offers a more accessible method for altering molecular levels in song control nuclei. Viral strategies have several advantages: First, stereotaxic viral injections offer ~100% viability. Second, viral injections enable temporal specificity as the encoded transcript will only become expressed following delivery of the virus into the nervous system. Finally, viruses allow for spatial specificity as certain viruses are expressed only in the region that has been injected. In those cases where a virus is anterogradely or retrogradely trafficked, this feature can be exploited in the service of circuit-mapping. In this regard, the nested arrangement of the song control nuclei within the songbird brain (Fig. 1) offers a particularly tractable circuit for genetic interrogation of behavior using targeted viral injections.

Groundbreaking work by Haesler et al. (2007) enabled the lentiviral-mediated expression of short hairpin (sh) RNA driven by the U6 promoter to knockdown the speech and language related transcription factor, Forkhead box P2 (FoxP2), in the basal ganglia nucleus Area X (Fig. 1). The results confirmed that, as in humans, the normal function of FoxP2 is required for vocal learning. In another use of lentivirus, Matsunaga et al. (2011) overexpressed the cell adhesion molecule cadherin7 in primary motor cortical nucleus, RA, of Bengalese finches (*Lonchura striata domestica*) and found that it also led to song learning deficits. It should be noted that RA is the output of the entire song circuit, and thus may be the song nucleus most sensitive to disruption. Yet, the deficits were modest in magnitude and required that a large amount of data be segregated due to low transfection rate. In the first use of optogenetics in birds, Roberts et al. (2012) used both adeno-associated virus (AAV) and herpes simplex virus (HSV) to overexpress channel-rhodopsin in the premotor cortical nucleus HVC. Disruption of HVC firing patterns during exposure to the song model disrupted song learning. Unfortunately, no information was provided about neuronal specificity, transfection rate, or pattern of transfection of these viruses other than that the non-specific cytomegalovirus (CMV) promoter was used for in vivo work. As detailed below, viruses similar in design and source to those used by Roberts et al. (2012) can be used to retrogradely transfect afferents to the song system. More recently, Yazaki-Sugiyama et al. (2015) used an AAV to express heteromeric glutamate and chloride channels from Caenorhabditis elegans in the primary motor nucleus, RA, of zebra finches. By providing the ivermectin inhibitory ligand, they were able to show that reversible silencing of a subset of RA neurons resulted in deterioration of the quality of song elements, but not in the timing or order with which these elements were sung.

Of the few studies that used stereotaxic viral injection to alter gene expression in songbirds, only Matsunaga et al. (2011) did so to overexpress a gene normally present in song control nuclei. We sought to test whether or not constitutively high levels of FoxP2 in Area X—like constitutively low ones—would be deleterious to song learning. Our rationale was based on the observation that Area X FoxP2 levels are regulated by singing and social context, being high when male birds are not singing or are directing their songs to females, but falling when males practice their songs alone (Teramitsu and White 2006; Miller et al. 2008; Teramitsu et al. 2010; Hilliard et al. 2012; Chen et al. 2013; Shi et al. 2013; Thompson et al. 2013). This behavior-linked regulation suggested that either constitutively low, as in Haesler et al. (2007), or high levels would similarly disrupt song learning. To that end, we tested a large panel of viral vectors for suitability in overexpressing genes in the song system. To do so would require a virus that fulfilled the following criteria, and for the following reasons:

1. The virus and associated promoter should transduce only neurons. *Rationale*: FoxP2 is expressed only in neurons.
2. The virus should transfect a large number of neurons. *Rationale*: Previous work in both zebra finches (described above) and mammals suggests that ≥ 20% should be the lower limit of transfection rate to achieve a behavioral effect (e.g., Rumpel et al. 2005).
3. Transfected neurons should express the transgene at a high level. *Rationale*: Weak transduction levels may not sufficiently override endogenous control mechanisms.
4. The virus should not retrogradely transfect afferent neurons. *Rationale*: Behavioral regulation of FoxP2 expression in the song system is limited to Area X and not its two afferent nuclei, HVC and LMAN.
5. Viral expression should not spill over into adjacent reasons. *Rationale*: Similar to the retrograde requirement, the interpretation of a behavioral phenotype should not be confounded by off-target over expression.
6. The virus should not damage the tissue or cause neurotoxicity. *Rationale*: Any effect of FoxP2 overexpression will ideally be studied in an intact circuit. Damage to song control nuclei will produce song deficits and thus confound interpretation of behavioral changes caused by the genetic manipulation.
7. The virus should provide persistent expression. *Rationale*: The sensorimotor critical period for song learning in zebra finches is ~60 days. Thus, the viral disruption should persist over this period.

Below, we describe our efforts to test a variety of viruses to identify a subset that met these criteria.

## Methods

### Subjects

All animal use was in accordance with NIH guidelines for experiments involving vertebrate animals and approved by the University of California Los Angeles (UCLA) Chancellor’s Institutional Animal Care and Use Committee and were consistent with the American Veterinary Medical Association guidelines. Birds were obtained from our breeding colony, and housed in climate-controlled rooms inside cages and aviaries with a 13:11 light/dark cycle including half hours of dawn and dusk lighting conditions. Birds had unlimited access to food, grit, and water and were provided both nutritional supplements (cuttlebone, spray millet, chopped hard-boiled eggs, orange and green vegetables, calcium supplements) and environmental enrichments (a variety of perches, swings, mirrors, nesting materials and water baths).

### Stereotaxic neurosurgery

Neurosurgeries were conducted on juvenile male zebra finches (~30 days of age) as previously described (Heston and White 2015). Surgery on adults (>100 days) followed a nearly identical procedure with the volume of injections ranging from 0.5 to 1.0 µl.

### Histological methods

To examine the efficacy of stereotaxic targeting and viral-driven protein expression, birds that received viruses expressing fluorescent reporter genes were overdosed with isoflurane inhalant and then trans-cardially perfused with warm saline followed by ice-cold 4% paraformaldehyde in 0.1 M phosphate buffer, and their brains were extracted for histological analysis. In cases where levels of native fluorescence provided by the reporter were weak, immunological enhancement was performed as previously described (Miller et al. 2008). Additional enhancement was provided by tyramide signal amplification (TSA) as previously described (Condro and White 2014).

## Results

### FoxP2 does not overlap with a marker for pallidal neurons

A large body of research seeking to understand the role of FoxP2 in mediating speech and language deficits has focused on its expression within striatal medium spiny neurons (MSNs). This is because major abnormalities in both humans and mice that carry FoxP2 mutations (Belton et al. 2003; Liegeois et al. 2003; Groszer et al. 2008) occur in the striatum, and MSNs are not only the principal cell type of the striatum but also the only striatal neuron type that expresses FoxP2. Interestingly, however, prior work had shown that *FOXP2* mRNA is enriched in the internal segment of the globus pallidus, early in human development, (GPi; Teramitsu et al. 2004). More recent work in rodents has revealed a unique population of FoxP2-positive neurons in the external segment of the globus pallidus (GPe) that send an ascending projection back to the striatum, have unique electrophysiological properties in vivo, and play a special role in stopping behaviors (Mallet et al. 2012, 2016). Thus, FoxP2 has the potential to be expressed in either the direct (likened to mammalian GPi) or indirect (likened to mammalian GPe; Farries et al. 2005) Area X pallidal cell population and play an important role in controlling behavior.

**Fig. 2:**
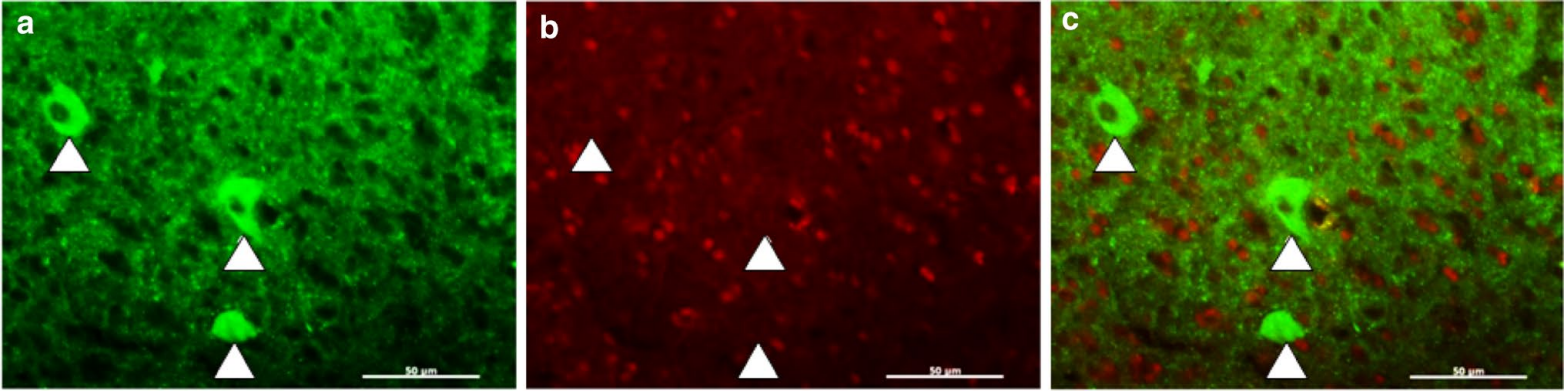
FoxP2 is not co-expressed in pallidal neurons labeled by anti-Lant6 antibody. **a** Anti-Lant6 antibody labels three large cells (*arrowheads*) in a tissue section containing Area X. **b** Anti-FoxP2 antibody signals appear enriched in nuclei that are distinct from the cells labeled in (**a**). **c** Merged images show no overlap between the pallidal marker Lant6 (*green*) and FoxP2 (*red*). *Scale bar* 50 uM

To be mindful of a potential pallidal population of FoxP2-positive cells in selecting a virus, we searched the literature for evidence of FoxP2 expression in Area X pallidal neurons. One report suggested that Area X pallidal neurons do not express FoxP2 (Rochefort et al. 2007) because pallidal neurons do not undergo neurogenesis, and newly born neurons express FoxP2. However, these two pieces of evidence still allow for the existence a non-neurogenic population of FoxP2-positive pallidal neurons. We thus evaluated any overlap between the expression of FoxP2 and the pallidal marker Lys8-Asn9-neurotensin8-13 (Lant6; Reiner and Carraway 1987). We failed to find overlap between these two markers in Area X neurons (Fig. 2). Although this result does not rule out the possibility of a small subset of FoxP2-expressing pallidal neurons (Abdi et al. 2015), a virus that tended not to transfect pallidal neurons was deemed preferable over one that did.

### Lentivirus

Based on the success of Haesler et al. (2007), the first category of virus tested was lentivirus, a class of vectors that is derived from the human immunodeficiency virus. Lentivirus is a retrovirus that incorporates its DNA into the genome of the host cell and, as a consequence, expression of this DNA is permanent in both dividing and non-dividing cells. The amount of DNA that can be delivered is relatively large (7–8 kb) and cloning capacity is thus not a major issue. Moreover, lentivirus exhibits a high degree of neural tropism.

**Fig. 3:**
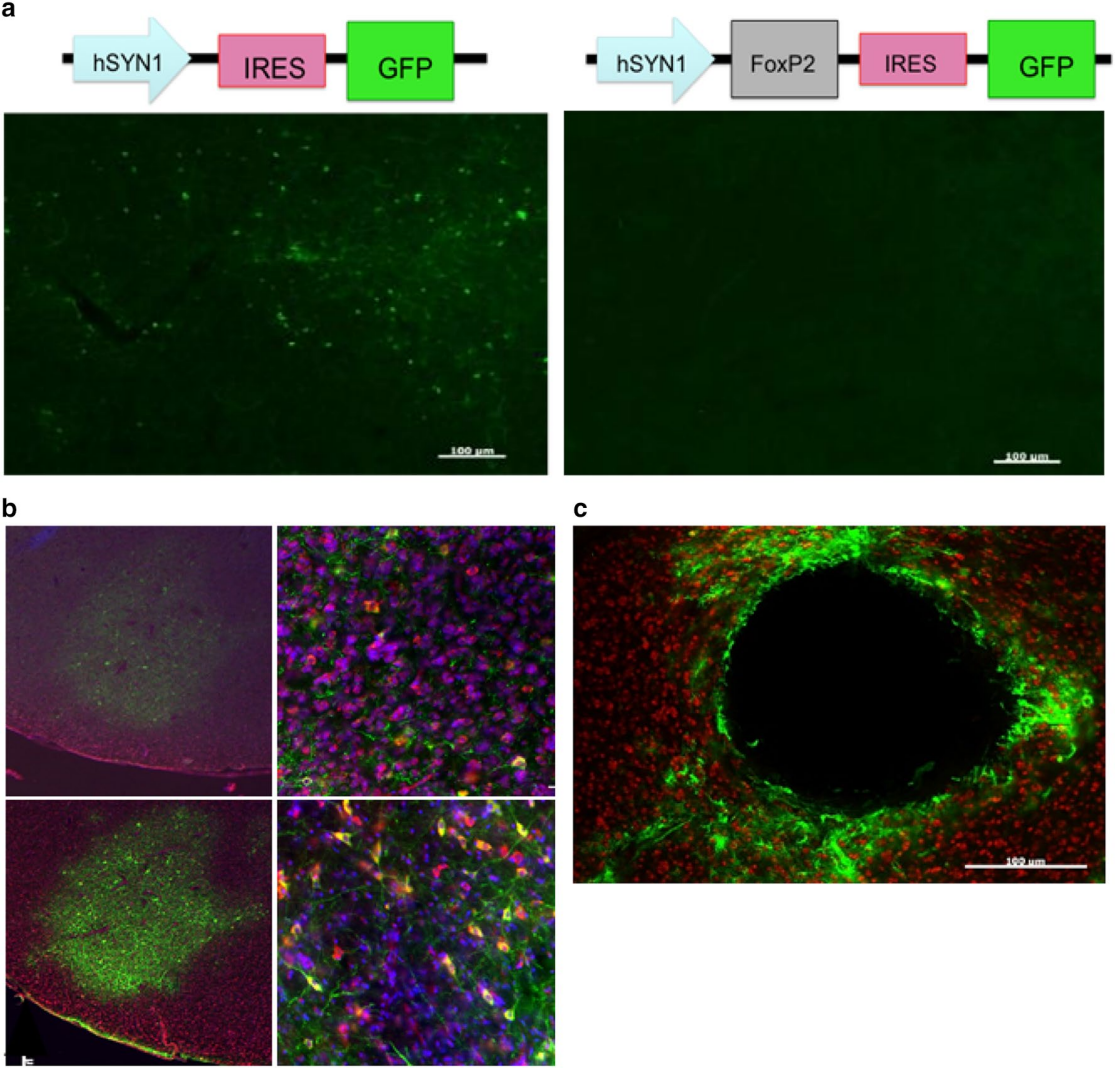
Characterization of three lentiviruses obtained from the UCLA viral vector core for their ability to transduce Area X neurons. **a** *Top* control (*left*) and experimental (*right*) constructs are depicted. *Below* photomicrographs show GFP expression levels following injection into Area X. These viruses achieved sparse GFP expression that could only be detected using a GFP antibody in sections that received the control construct (e.g., *left panel*). Little to no GFP was observed in the contralateral hemisphere injected with the experimental virus, despite immunological enhancement with an anti-GFP antibody (*right panel*). *Scale bar* 100 uM. **b** Photomicrographs of Area X from a bird brain injected with ultra-centrifuged hSyn1 lentivirus. GFP is shown as *green*. The tissue was counterstained for NeuN (*red*) and DAPI (*blue*). No fluorescence was detected without an anti-GFP antibody. *Top left image* shows GFP positive signal following conventional immunohistochemical enhancement anti-GFP antibody. A higher magnification of the same section (*top right*). *Bottom images* show enhancement of GFP signal following TSA amplification. Despite enhancement, the infection was sparse with few GFP-positive cell bodies. **c** Photomicrograph shows lesion site in Area X following injection of lentivirus containing the PGK promoter. *Scale bar* 100 μm

Two lentiviruses were obtained from the UCLA viral vector core. Both used the human synapsin1 promoter (hSyn1) to drive transgene expression. This promoter had been demonstrated to exclusively transduce neurons (Kügler et al. 2003; Shevtsova et al. 2005), consistent with the fact that synapsins participate in the regulation of exocytosis at neuronal synapses. The experimental virus had the design hSyn1-FoxP2-IRES-GFP, whereas the control virus had the design hSyn1-IRES-GFP (Fig. 3a). The major problem with these viruses was that they transfected too few Area X neurons. In addition, they expressed very low levels of GFP that could only be detected using an anti-GFP antibody and TSA. In fact, GFP was barely detectable in hSyn1-FoxP2-IRES-GFP injected tissue that had undergone TSA amplification. Because the FoxP2 and GFP were translated separately by virtue of the IRES sequence, GFP levels did not necessarily reflect the expression levels of FoxP2. Nevertheless, the results were not promising and this was abandoned.

We tested an additional set of lentiviruses from the UCLA Core. One of these used the hSyn1 promoter to express channel-rhodopsin covalently attached to enhanced yellow fluorescent protein [ChR2-eYFP; courtesy of J. Feldman (UCLA) and described in Pagliardini et al. (2011)]. Several similarities and differences exist between this and the previous lentiviruses. The major similarities are that both were produced by the same viral core and used the same promoter. One major difference was that the design of the construct was such that the molecule of interest, ChR2, was covalently attached to the fluorophore, eYFP that was used to detect expression of the virus. Thus, one would expect a 1:1 ratio of both molecules, which may not be the case in viruses that utilize an IRES sequence. Second, as a consequence of being covalently attached to ChR2, which encodes a membrane ion channel, the fluorophore was expressed in the cell membrane rather than being free to fill the cytosol. Because neurons in general, and particularly small neurons such as MSNs, have high surface area-to-volume ratios, levels of this virus could potentially appear greater than would a comparably transfected cell in which the fluorophore fills the intracellular compartment. Finally, this virus was ultra-centrifuged to increase the titer. The titer was unknown but because it was obtained from the same viral core as the previous lentivirus it would almost certainly be at a higher titer.

Upon initial inspection of the injected brain region, no transduction was apparent (data not shown), but expression was revealed using an anti-GFP antibody (which detects multiple fluorophores including YFP) and further enhanced by TSA amplification (Fig. 3b). This virus clearly was able to transduce Area X neurons, but had the following limitations: It transfected a relatively diffuse population of cells; the level of transfection was so low that several amplification steps were necessary to reveal a signal, and; both of these issues would have likely been worse had the virus not been ultra-centrifuged. For these reasons and others related to the previous lentiviruses, the hSyn1-lentivirus approach was abandoned.

Finally, a phosphoglycerate kinase promoter (PGK) driving GFP expression was tried as this glycolytic enzyme is ubiquitous to all cell types (Chiarelli et al. 2012). This virus was found to be the least successful of all the lentiviruses tested as it created a large hole at the injection site and most of the signal appeared due to auto-fluorescent scar tissue rather than to expression within neurons (Fig. 3c).

Lentivirus has the strength of persistent expression and, with an appropriate promoter, may be capable of strongly transducing avian neurons. Barring the PGK lentivirus, there was no evidence of neurotoxicity or off-target transfection. Lentivirus did not appear to travel retrogradely nor spill into adjacent tissues, thus allowing focal transfection of brain nuclei. Its major flaw was its inability to transfect large numbers of neurons. One possible strategy to overcome this hurdle would be to concentrate the virus via ultra-centrifugation. Even this, however, might not be viable strategy as the hSyn1-ChR2-eYFP virus was ultra-centrifuged and, while this did improve transfection rate, it was still insufficient to reach our criterion of ≥ 20% required to move forward with behavioral testing. On the other hand and as noted above, others have used lentiviruses in zebra finches, including in Area X, and have not only reported higher levels of transfections but have altered song behavior, so these conclusions may better be limited to lentivirus obtained from the UCLA Viral Core rather than to lentivirus generally. As will be demonstrated with adeno-associated virus (AAV; see below), the source of the virus can be a critical but underappreciated variable (R. Neve, Massachusetts Institute of Technology, personal communication).

**Fig. 4:**
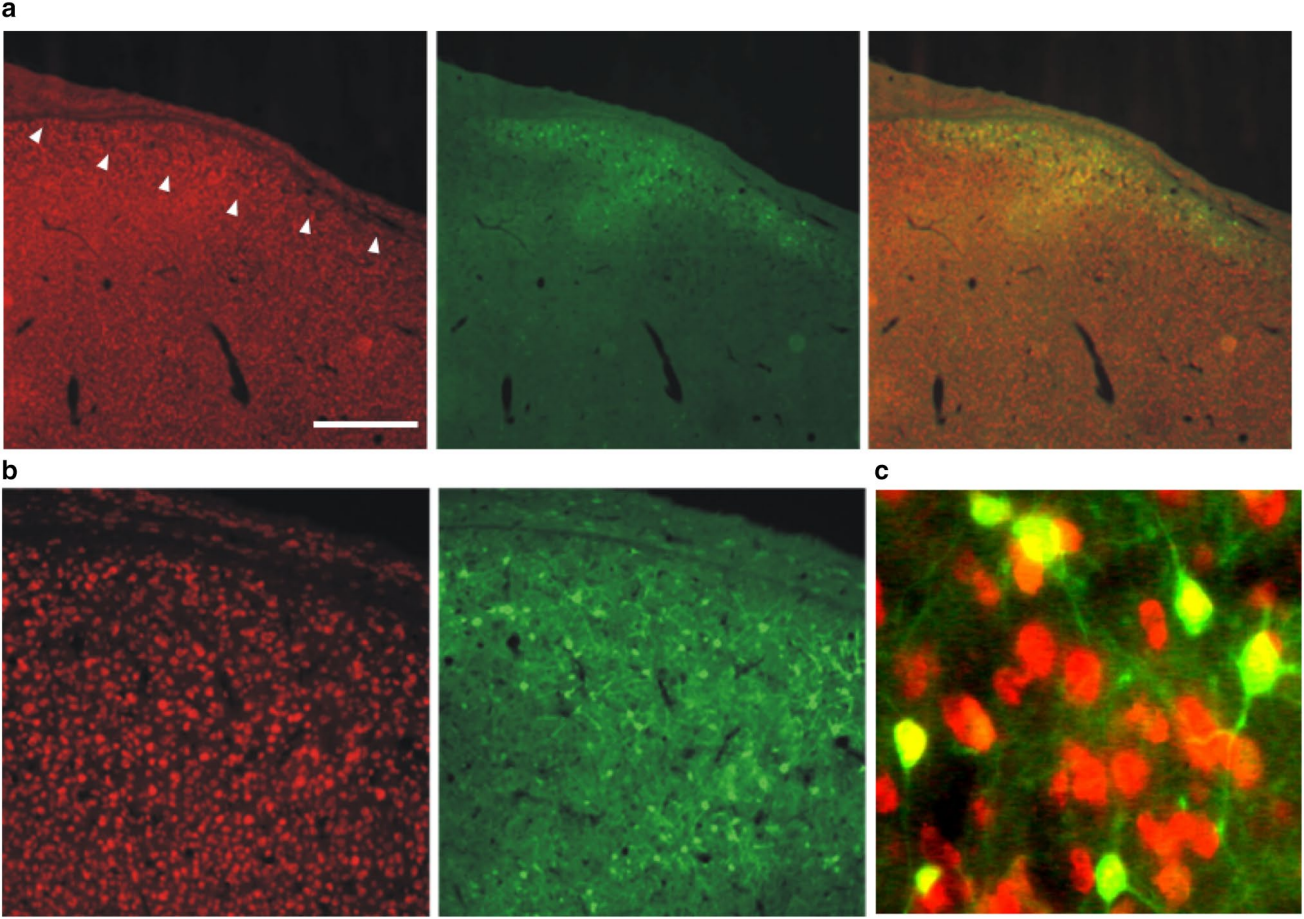
AAV hSyn1 GFP transduces HVC neurons. Photomicrographs show NeuN-positive neurons (*red; right panels*), GFP reporter signals (*green; middle panels*) and merged images (*left panels*). **a** Low magnification images show HVC (*arrowheads* denote ventral border) as the slight increase in neuronal density lying just superficial to the thin hyperpallial layer. *Scale bar* 500 μm. **b** Higher magnification is shown in *left and middle panels*. **c** Merge of even higher power image illustrates that only NeuN positive cells are transduced

An additional lesson learned in the foray into lentivirus is that the hSyn1 promoter does not appear to work well in Area X of zebra finches and requires amplification to detect transfected cells. One possible explanation is that zebra finches apparently lack the synapsin 1 gene (Warren et al. 2010) and thus its promoter may be non-functional or lead to only low levels of gene expression. This conclusion should be tempered, however, as we were able to observe hSyn1-driven GFP expression in the premotor song control nucleus, HVC, using an AAV (Fig. 4; courtesy of Sebastian Kügler, U Göttingen). Similarly, Tanaka et al. (2016) successfully used lentivirus and the hSyn1 promoter to drive overexpression of mutant Huntingtin in Area X. These results indicate that hSyn1 promoter can transduce neurons in the zebra finch song circuit. Nevertheless it is possible that this promoter is sub-optimal and that higher levels of expression could be achieved with a different promoter.

### AAV

The second class of virus tested was AAV. Its DNA incorporation is episomal but expression can persist for years in non-dividing cells. Based on an informal survey of the literature, AAV seems to be the most commonly used virus for manipulating behavior. One drawback is the limited cloning capacity which is ~4.7 kb. One advantage is that the viral tropism can be altered by exploiting the natural capsid diversity of isolated serotypes. For example, AAV serotype 2 uses heparin sulfate proteoglycans to enter cells, whereas AAV5 also binds to N-linked sialic acids and platelet-derived growth factor receptors. Cross-packaging the genome of one serotype with the capsid of another (e.g. AAV2/5) can help to tailor the expression to different sets of cells (Shevtsova et al. 2005). We first tested an AAV5, which is not neuron-specific, to drive expression of GFP off of the hSyn1 promoter (Fig. 4). The reason for testing this promoter was to see if it drove neuron-specific expression despite widespread transfection of the virus. While we observed robust transfection of HVC neurons, the same virus led to only low levels in Area X (data not shown). This difference likely reflects the different cell types in each region, with a predominance of inhibitory neurons in Area X, in contrast to HVC.

**Fig. 5:**
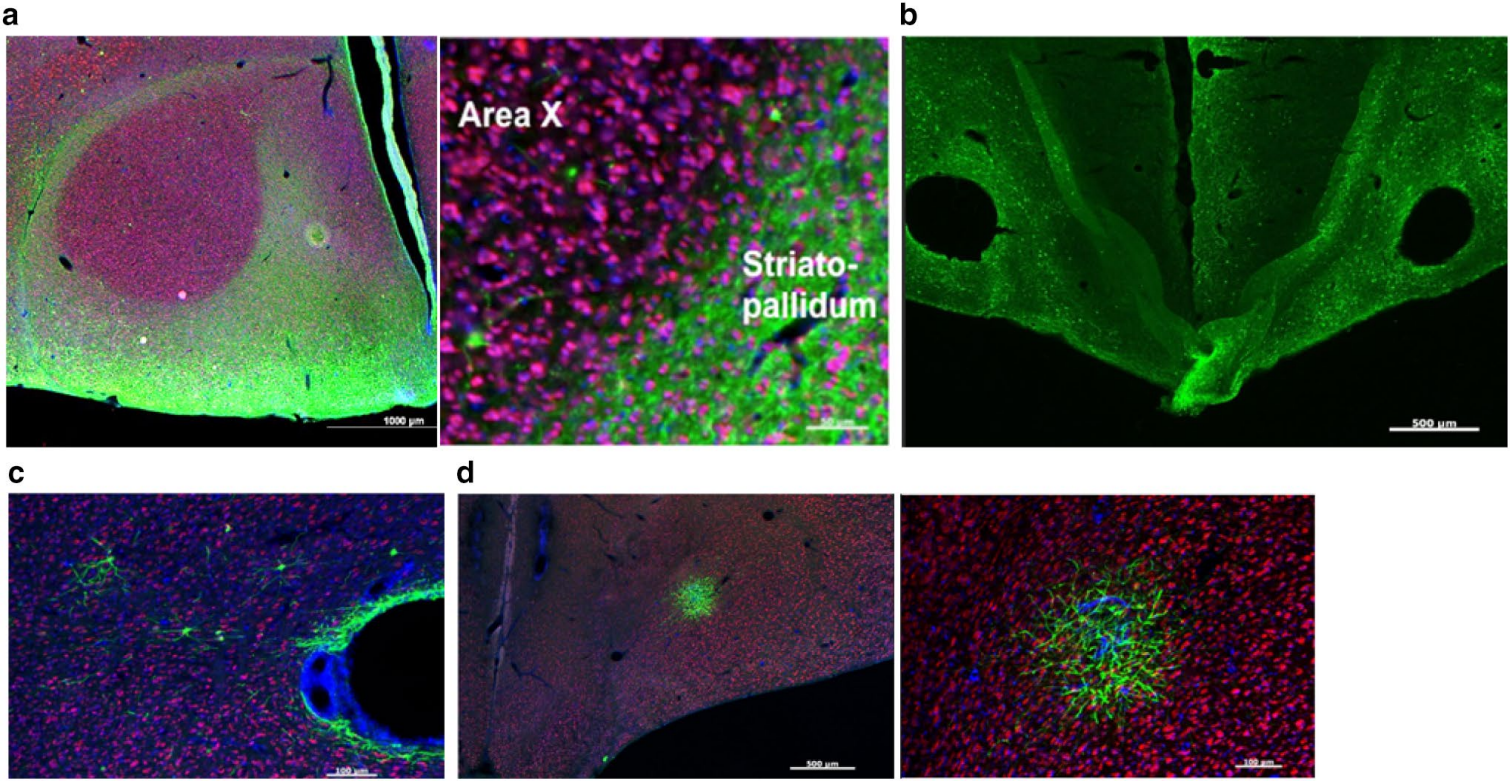
Characterization of four AAVs obtained from the U Penn viral vector core for their ability to transduce Area X neurons. In *all panels, green signals* show GFP, *red signals* show NeuN, and *blue signals* show DAPI staining. **a** *Left photomicrograph* reveals that AAV2/5 transfects large portions of the brain except for Area X. *Scale bar* 1000 μm. *Right* higher magnification shows robust transduction in the striatopallidum outlying Area X. *Scale bar* 50 μm. **b** AAV2/1 shows robust transduction but also induces lesions at the injection site. *Scale bar* 500 μm. **c** Representative photomicrograph shows that AAV2/rh10 has a low transfection rate in Area X and induces a lesion at the injection site. *Scale bar* 500 μm. **d** *Right photomicrograph* shows low levels of transduction by AAV2/8. *Scale bar* 500 μm. Even at higher magnification (*right*), there were very few GFP-positive cell bodies in the most densely transfected region. *Scale bar* 100 μm

We next tested a panel of viruses from the University of Pennsylvania (U Penn) viral core that used the CMV promoter to drive expression of GFP. As a group, all of these viruses failed in meeting the full set of criteria and several of them failed in very interesting ways. For example, when injected into Area X, AAV2/5 drove expression throughout most of the brain with one notable exception-Area X itself. Indeed, the outline of Area X is clearly observable as the negative space created by off target GFP expression (Fig. 5a). In another bird injected with this virus, a salt-and-pepper pattern of expression was observed throughout the brain (data not shown). Again, low levels of expression were seen at the injection site, but unlike the other bird injected with this virus, it did not create the negative image of Area X. Instead, expression levels were uniformly low in the striatopallidum (data not shown). Thus, it is unclear whether the “negative image” pattern is a reproducible effect that could be harnessed in interesting ways (i.e. comparing the FoxP2 overexpression in Area X versus the outlying striatopallidum) or whether this was due to idiosyncrasies of that particular surgery. In either case, AAV2/5 showed an inconsistent pattern that was not suitable for the project outlined here.

Other AAV from this panel failed in less interesting and perhaps less telling ways. For example, AAV2/1 showed bilateral lesions at the site of injections and GFP-positive cells were broadly distributed across the brain (Fig. 5b). A similar pattern was observed with AAV2/rh10 although it showed a somewhat lower transfection level (Fig. 5c). AAV2/8, showed a similar issue with lesions at the injection site but unlike AAV2/1, GFP-positive cells appeared limited to the region immediately surrounding the injection/lesion site (Fig. 5d).

**Fig. 6:**
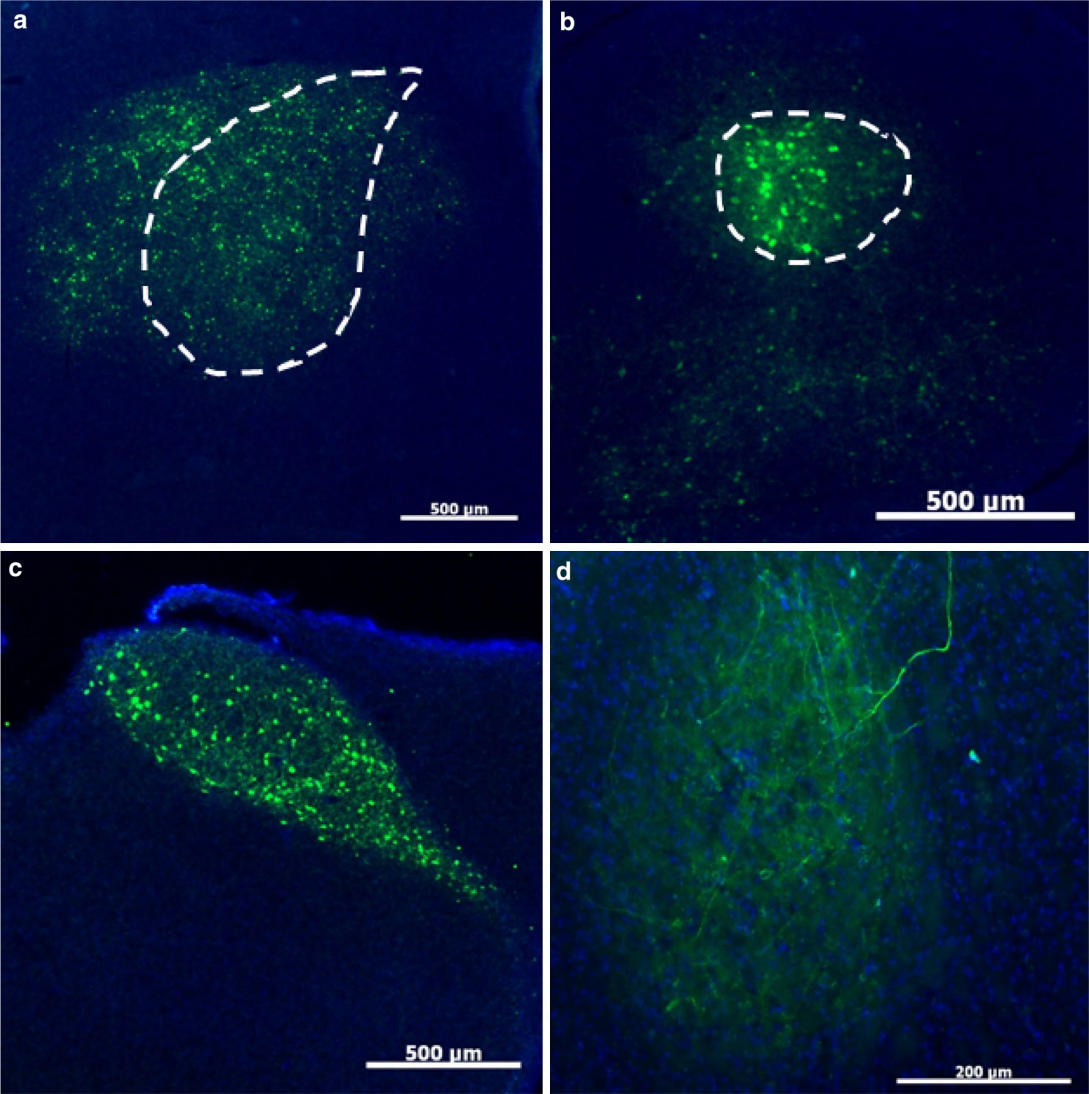
U Penn AAV2/1-CB7-GFP retrogradely infects at least one, if not more, Area X afferent nuclei. **a** High numbers of GFP-positive cells are visible at the injection site in Area X (*dotted outline*). *Scale bar* 500 μm. **b** High numbers of YFP-positive cells (effectively labeled by an anti-GFP antibody) were also identified in LMAN (*dotted outline*). *Scale bar* 500 μm. **c** The near perfect fill of HVC is best explained by retrograde transfection from Area X. *Scale bar* 500 μm. **d** Retrograde transfection was further confirmed by expression of GFP-positive fibers in RA, which does not receive input from Area X but does receive input from LMAN and HVC. *Scale bar* 200 μm

One additional virus from U Penn that was not from the original panel, failed but ultimately proved very useful in identifying elements of a successful virus. This was an AAV2/1 driving expression of CB7-GFP. The CB7 promoter is a CMV and chicken beta actin fusion promoter similar to the more commonly used CAG promoter. It should also be noted that while this virus was obtained from the U Penn Viral Core, it was ordered several years later than the previously described U Penn AAV so it is unclear whether the differences between this and previous viruses are due to differences in the viral construct or non-trivial differences in the way this viral core has improved viral production. This virus fulfilled every requirement except that it traveled retrogradely. This can be seen in Fig. 6 in which there is strong GFP expression in Area X, but also in cell bodies in LMAN (Fig. 6b) and HVC (Fig. 6c) both of which project to Area X. Because LMAN is so near Area X and the injecting electrode passes through LMAN on its way to Area X, it was unclear whether this off-target expression was the result of spillover or of retrograde transfection. Spillover was ruled out by expression of GFP in HVC which is ~7 mm dorso-caudal to Area X. There were even GFP-positive terminals in RA which, like Area X, receives synaptic input from HVC (Fig. 6d). These GFP-positive terminals likely came from LMAN as the Area X-projecting LMAN neurons send bifurcating axons to RA, whereas separate populations of HVC neurons send projections to Area X or RA.

**Fig. 7:**
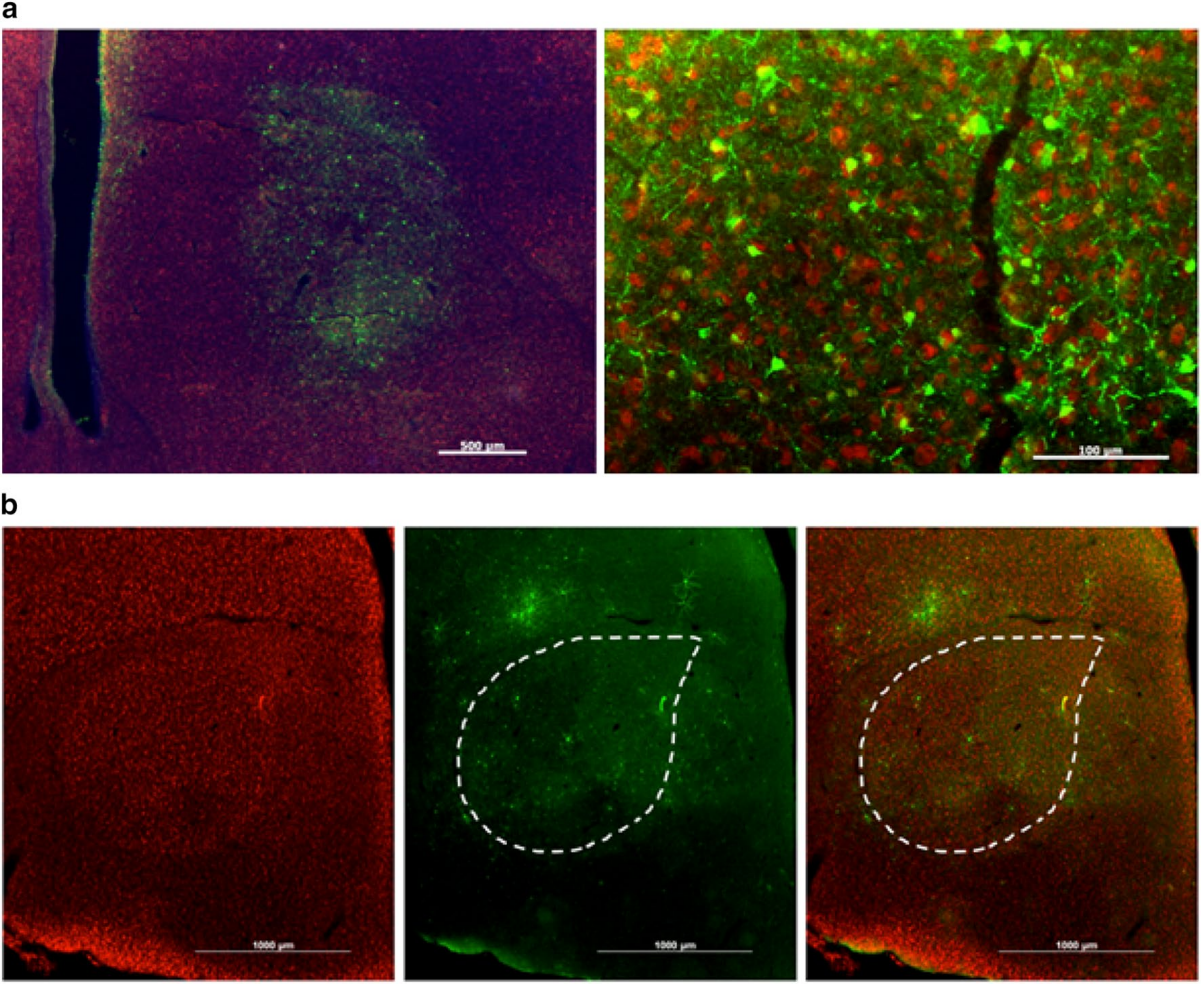
Virovek AAV2/5 is effective in transducing Area X neurons, whereas AAV2/9 is not. **a** Low magnification photomicrograph (*left*) shows that AAV2/5 can transfect a delimited region of the brain. Some transfection is seen dorsal of the striato-nidopallial border, likely the result of mis-targeting of the injection. *Scale bar* 500 μm. Higher magnification, *right*, reveals a high transfection rate. *Scale bar* 100 μm. **b** Anti-NeuN antibody signals (*red*) reveal Area X in left-most panel that is outlined in the *middle* and *right panels*. AAV2/9 injection in Area X leads to GFP-positive signals (*green, middle panel*) in cells that are diffusely scattered around the injection site. Images are merged in the *right panel*

Finally, we tested a panel of viruses from Virovek, a private company based out of Hayward, CA. Each of these viruses used a CMV promoter to drive expression of GFP, the same design of the AAV tested from U Penn, and several viruses of the same serotype as the U Penn panel. Thus, these viruses were of the same construct design and serotypes but differed only in where they were produced. By and large, the entire panel of Virovek AAV met most, if not all, of the criteria. All of the viruses showed strong transduction and the ability to confine expression to Area X. None of them showed any sign of transfection of off-target cells types, neurotoxicity, or retrograde trafficking. The primary difference was in the number of cells transfected by the virus. AAV2/1 and AAV2/5 (Fig. 7) transfected the largest number of cells with no clear difference between the two. The viruses from Virovek which did fail, such as AAV2/9 (Fig. 7) tended to do so by having low transfection rates and a diffuse pattern of expression which was neither confined to Area X but not brain-wide like some of the U Penn AAV.

**Fig. 8:**
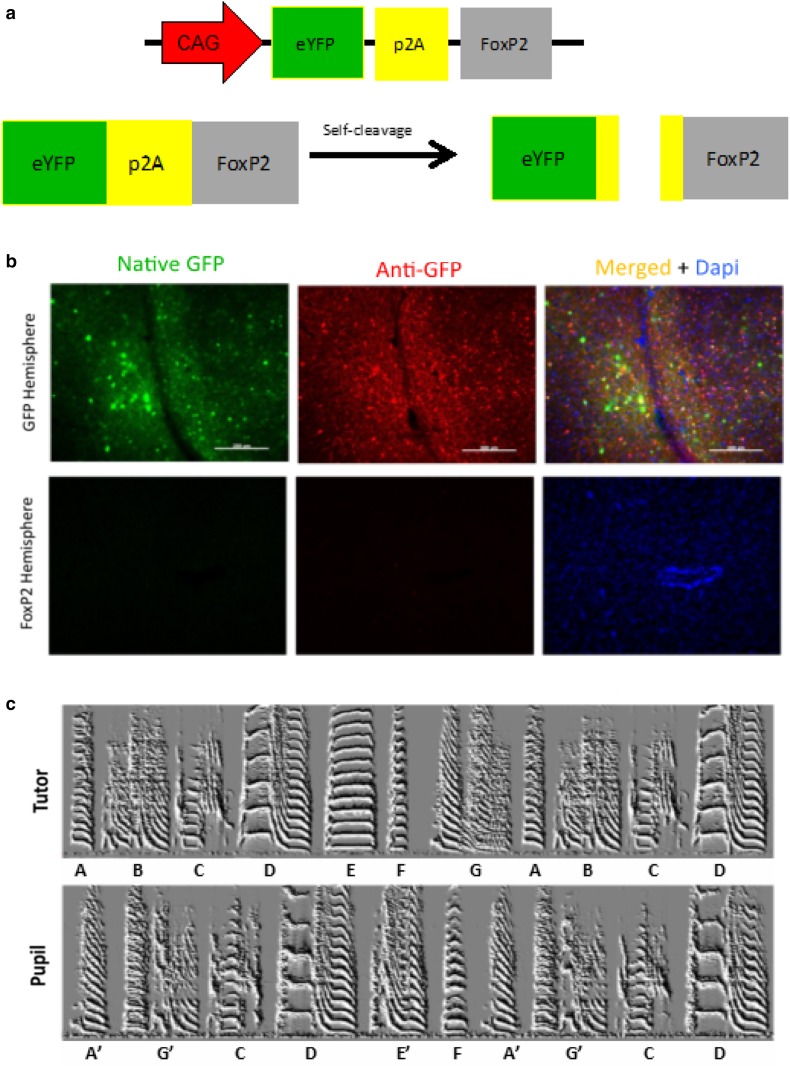
A p2A sequence is ineffective in expressing either GFP or FoxP2. **a** Schematic of virus that utilizes a p2A. In theory, a single gene product should be translated which creates a GFP-p2A-FoxP2 fusion protein that is then self-cleaved into separate GFP and FoxP2 molecules that each carry residual p2A peptides on their C- and N-termini, respectively. **b** *Top panels* the GFP virus-injected hemisphere showed high levels of native GFP (*green*) which was further enhanced using an anti-GFP antibody (*red*). *Lower panels* in contrast no GFP, native or enhanced, was visible in the contralateral hemisphere injected with the GFP-p2A-FoxP2 virus. *Blue* signals reflect DAPI staining. **c** Exemplar song spectrograms of an adult male tutor (*top*) and his adult (>100 days) male son (*bottom*)

As an additional exploration of AAV’s ability to overexpress genes, one final permutation was tested. Having settled on AAV2/1 from Virovek with a CAG promoter as the means by which to overexpress FoxP2 in Area X, we tested whether the limited cloning capacity of AAV could be overcome to express two molecules, FoxP2 and GFP, with a single virus using a p2A sequence (Fig. 8a). This peptide sequence, 18–22 amino acids long, would sit between FoxP2 and GFP in a large GFP-p2A-FoxP2 fusion protein, but would recruit a self-cleaving mechanism that would split it into a GFP molecule with about 10 amino acids of the p2A on its C-terminal and FoxP2 which had about 12 amino acids on its N-terminal.

The results using this virus were poor. First, essentially no GFP was detected in a bird unilaterally injected with the GFP-p2A-FoxP2 virus (Fig. 8b). This was in contrast to easily detectable levels of GFP expression of a virus expressing only GFP in the opposite hemisphere. Moreover, there was behavioral evidence that FoxP2 was not being overexpressed. Overexpression of FoxP2 leads to poor song imitation (Heston and White 2015). In contrast, a bird bilaterally injected with the GFP-p2A-FoxP2 virus showed normal song learning (Fig. 8c). Together, these results suggest that neither FoxP2 nor GFP were being overexpressed by this virus.

Depending on the serotype, promoter, and viral core, AAV can fulfill all of the required criteria. The U Penn viral core had a number of issues, but AAV from Virovek had a number of advantages. Several viruses transfected a large number of neurons with the exception of a few serotypes and none transfected non-neuronal cell types, an observation consistent with AAV’s high neuronal tropism in mammals. AAV also did not appear to be neurotoxic with the exception of a subset of early tested AAV obtained from U Penn.

Virovek’s AAV was the most promising and thus ultimately selected for overexpressing FoxP2. AAV2/1 and AAV2/5 were performed the best and no major difference was apparent. Ultimately, AAV2/1 was chosen, in part, because in rodents while both AAV2/1 and AAV2/5 have equal transduction efficiency in the striatum, AAV2/1 has a considerably lower efficiency in the pallidum. Given the lack of evidence of FoxP2 expression in Area X pallidal neurons (Fig. 2), a tropism against these neurons was viewed as an advantage.

Additionally, the U Penn virus that traveled retrogradely could prove useful for circuit mapping, circuit manipulation, or selective targeting anatomically defined cell populations. For example, a retrograde acting AAV could be injected into either Area X or RA and another AAV carrying a CRE-dependent gene could be injected into HVC. This would allow for selective targeting of Area X projecting neurons in HVC.

### HSV

The final class of viruses tested was herpes simplex virus (HSV). Like lentivirus and AAV, HSV has a high degree of neural tropism. It also has by far the largest cloning capacity of the three (Neve et al. 2005). The major and well-characterized limitation of HSV is that its expression is only transient. More specifically, there are two types of HSV both of which show different patterns of transient expression. Short term HSV (ST-HSV) has been described to drive only transient expression which begins several hours post-delivery but is gone by 8–10 days. In contrast, long-term HSV (LT-HSV) shows the same pattern of transient expression, but also retrogradely transfects afferent neurons and expression in these cells is thought to be permanent (personal communication, R. Neve). While neither expression pattern is suitable for constitutively elevating FoxP2 during development, it could be suitable for other uses that require only acute elevation.

**Fig. 9:**
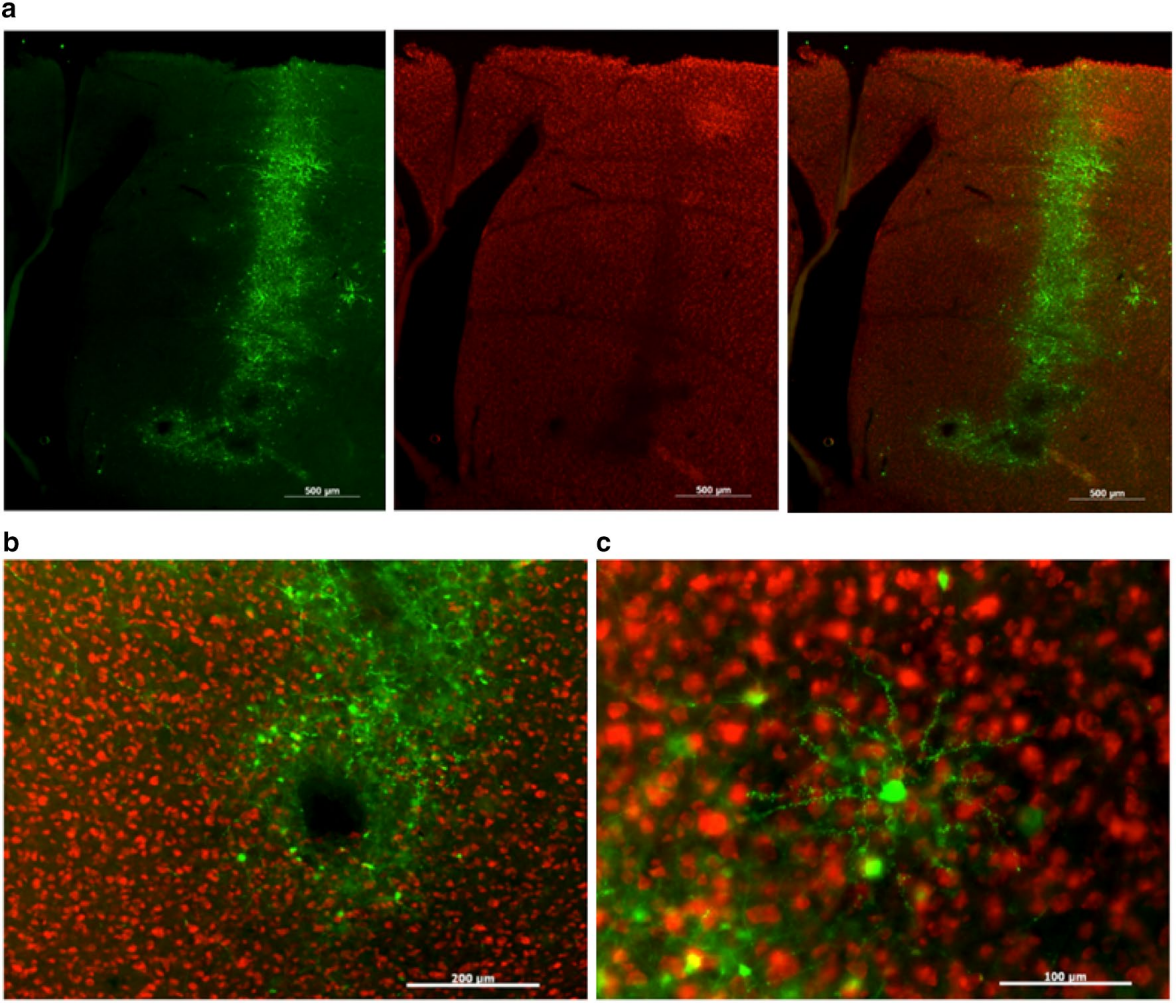
HSV effectively transfects Area X but can cause neurotoxicity. **a** Photomicrographs show high levels of GFP (*green* in *left* and *right panels*) along the injection track. A NeuN stain (*red signals, center* and *right panel*) reveals a darkened area indicative of neurotoxicity. *Scale bar* 500 μm **b** The HSV injection produced a relatively small lesion at the injection site. *Scale bar* 200 μm. **c** Higher magnification shows transduction of a neuron that morphologically resembles MSNs. *Scale bar* 100 μm

**Fig. 10:**
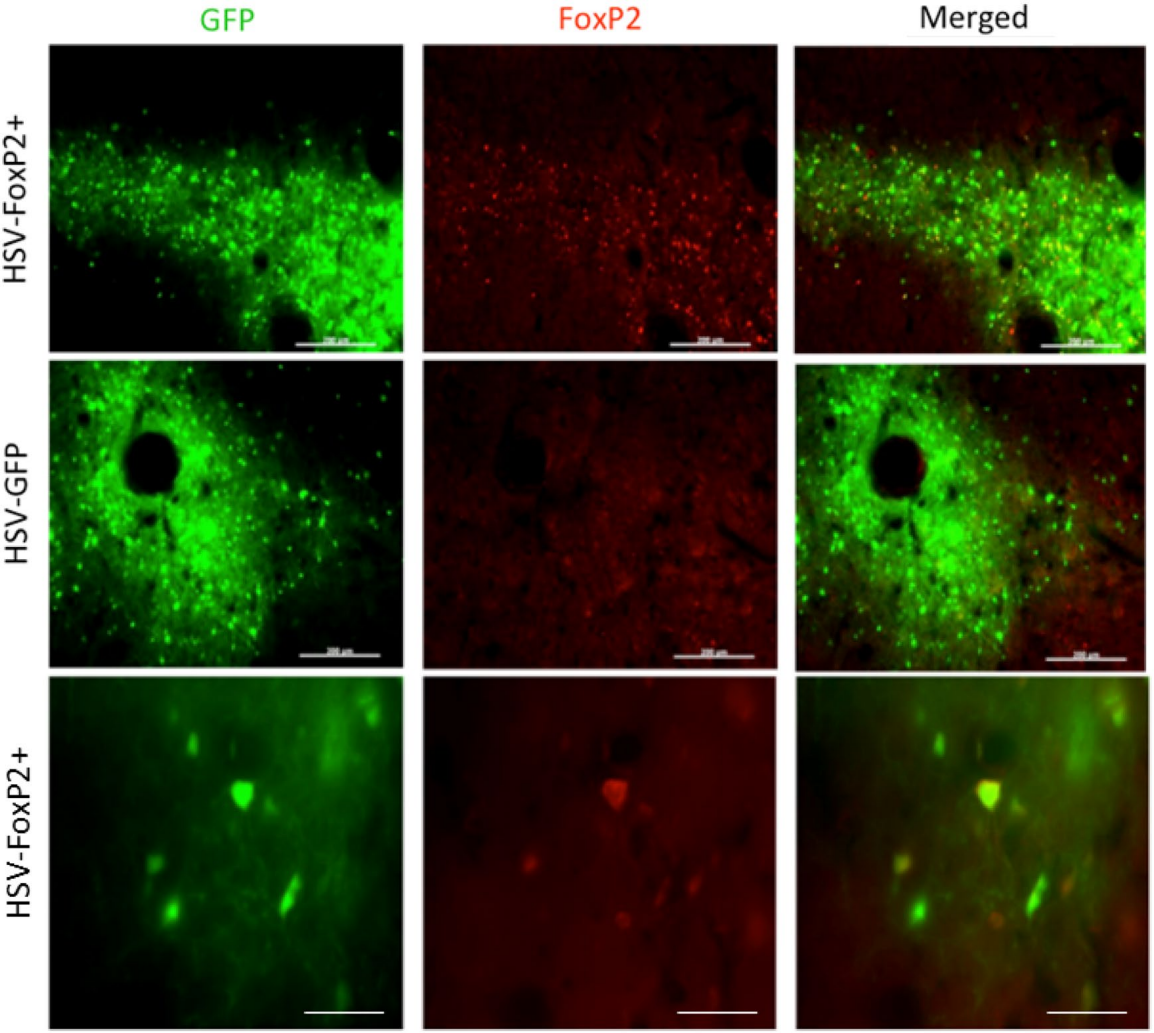
HSV is effective in overexpressing FoxP2. Photomicrographs from a bird that was injected with HSV-FoxP2+ in Area X of one hemisphere (*top* and *bottom rows*) and HSV-GFP in the other (*middle row*). While GFP is observed in both hemispheres, elevated levels of FoxP2 (*red signals*) are detected only in the HSV-FoxP2+ hemisphere and only in the region showing GFP. The tight correspondence between GFP and FoxP2 signals in the HSV-FoxP+ hemisphere is clear at higher magnification (*bottom* row). *Scale bars* 200 μm in *top two rows* and 50 μm in the *bottom row*

The first HSV tested was a LT-HSV which expressed GFP using the non-specific IE 4/5 promoter. When tested at full strength, this virus caused cell death (Fig. 9). Moreover, there was evidence of retrograde trafficking (data not shown). Fortunately, both issues could be mitigated, if not eliminated, by diluting the virus with saline prior to injection. To test whether the large cloning capacity of HSV could be exploited to overexpress two molecules (FoxP2 and GFP), we obtained a HSV which overexpressed FoxP2 with the IE 4/5 promoter and GFP with a CMV promoter. This virus showed high levels of co-expression of both genes (Fig. 10). In sum, the diluted HSV showed several desirable qualities including a high transfection rate, strong transduction of neurons, and the ability to transfect a brain region without spilling over into adjacent regions.

## Discussion

**Fig. 11:**
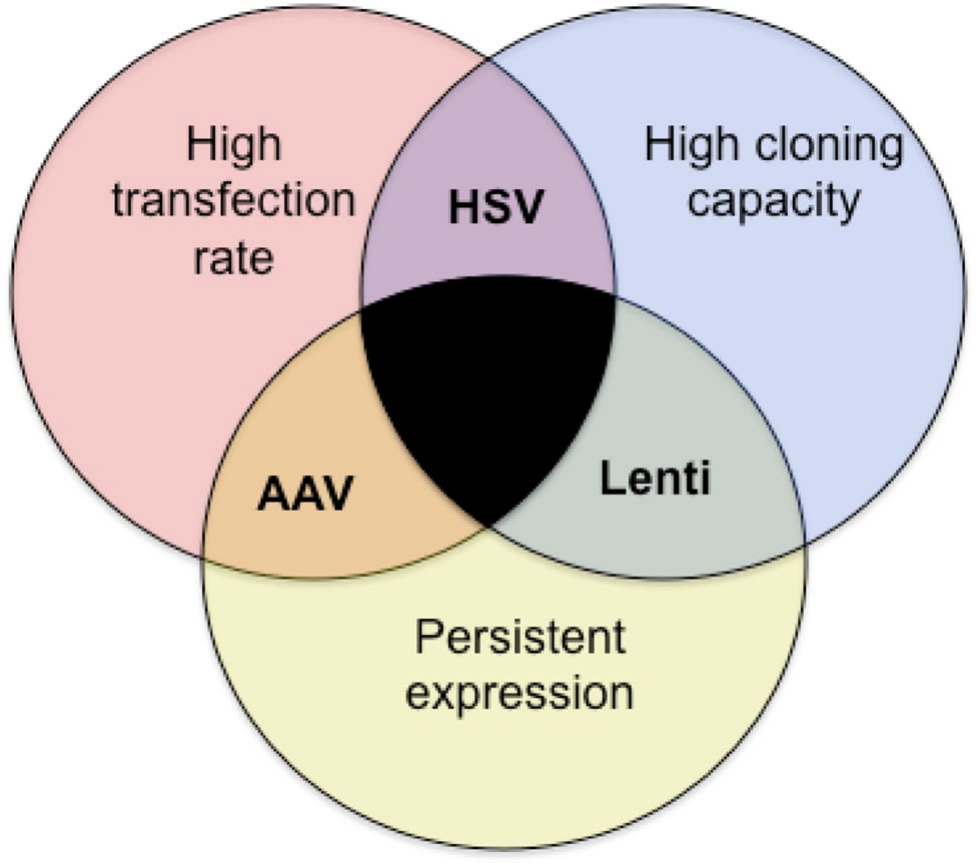
Venn diagram highlights the strengths and limitations of each virus type as they relate to specific requirements necessary for a given scientific application. Those requirements are (1) a high rate of transfection, (2) a high cloning capacity and, (3) persistent expression. Each viral type tested here fulfills only two requirements

Here, we qualitatively describe efforts to characterize various viruses in terms of their suitability for overexpressing genes, FoxP2 in particular, in Area X. Table 1 provides a summary of the efficacy of all the viruses that we tested. Ultimately, Virovek AAV2/1 using a CAG promoter was chosen as the most suitable for this task. Bilateral injections of ~100 nl into Area X of juvenile birds resulted in ~25% increase in FoxP2 protein, and disrupted song learning (Heston and White 2015). As a more general principal, each virus has its advantages that can be weighed against its disadvantages depending upon the scientific question. High transfection rate, high cloning capacity, and persistent expression are often desirable for genetic manipulation aimed at altering behavior (Fig. 11). Lentivirus, AAV, and HSV each fulfill only two of these criteria. Lentivirus has a high cloning capacity and persistent expression, but a low transfection rate. This virus may be most suitable when a large transgene must be expressed, but where a behavioral phenotype is not required. One such application is in electrophysiological or neuromorphological experiments designed to study cell autonomous effects. AAV transfects large numbers of neurons and does so in a persistent manner, but its cloning capacity can be a limiting factor. AAV is useful in obtaining a behavioral effect but may not be up to the task for expressing large genes or multiple genes. HSV transfects large numbers of neurons and has a large cloning capacity, but its expression is transient. Moreover, neurotoxicity and retrograde trafficking are a concern when using this virus but both appear to be mitigated by diluting the virus.

**Table 1:** Summary of the efficacy of viruses tested

Viral type	Serotype	Source	Promoter	Estimated transfection rate	Level of transfection (per cell)	Degree of spread	Neuronal specificity	Other notes
AAV	1	U.Penn	CMV	+++	++	+++	+++	Lesions at injection site
AAV	5	U.Penn	CMV	++	++	+++	+++	Showed tropism against transfecting area X
AAV	S	U.Penn	CMV	+	++	+	+++	Lesions at injection site
AAV	Rh/10	U.Penn	CMV	+	++	+	+++	Lesions at injection site
AAV	1	U.Penn	CBA	+++	+++	++	+++	Retrogradley transfected afferents
AAV	1	MIT/Virovek	CMV	+++	+++	++	+++	
AAV	2	MIT/Virovek	CMV	++	+++	+	+++	
AAV	5	MIT/Virovek	CMV	+++	+++	++	+++	
AAV	8	MIT/Virovek	CMV	++	+++	++	+++	
AAV	9	MIT/Virovek	CMV	++	+++	++	+++	
Lentivirus	n/a	UCLA viral core	Synapsin 1	+	+	+	+++	
Lentivirus	n/a	UCLA viral core	CMV	+	+++	+	+++	
Lentivirus	n/a	UCLA viral core	PGK	+	+++	+	+	Caused lesion; infected non neuronal cell types
HSV	ST-HSV	MIT/Virovek	CMV	++	+++	++	+++	Requires dilution to avoid toxicity and retrograde transfection

Estimated transfection rate: + = <5%, ++ = 5–20%, +++ = >20%

Level of transfection per cell: + = not visible without IHC, ++ = visible but greatly enhanced by IHC, +++ = easily visible, not enhanced by IHC

Degree of spread: + = transfects an area much smaller than Area X, ++ = transfects an area much comparable in volume Area X, ++ + = transfects an area much greater than Area X

Neuronal specificity: + = transfects non-neuronal cell types exclusively, ++ = transfects neuronal and non-neuronal cell types, +++ = transfects neuron exclusively

In addition to these concerns, there are practical issues. For example, AAV is biosafety level one, whereas lentivirus and HSV are biosafety level two, rendering the former easier to work with. An as yet unanswered question that is worth addressing is whether the CRE-lox system is effective in birds. In theory, it could be harnessed for combinatorial strategies aimed at transfecting discreet cell populations. It should not, however, be taken for granted that this will work, as the exemplars shown here illustrate that such viral tools must be empirically validated in the model organism of choice. Although not shown here, it is worth mentioning that results achieved in vivo are not always mirrored in vitro, e.g. in cell culture systems. Anecdotally, we found that lentivirus was more effective at transducing cultured neurons from hatchling zebra finch telencephali, than it was in vivo. AAV exhibited the opposite trend. This may reflect the different ages of the tissues involved, or a change in the components of cell membranes that occurs during the culturing process. Moving forward, it may be useful to identify additional viral types that can be characterized with the aim of providing more specific types of tropism, and possibly achieve all three goals (Fig. 11) of high cloning capacity, high transduction levels and persistent expression. Indeed, a recent study (Dimidschstein et al. 2016) describes a novel recombinant AAV that successfully transduces GABAergic interneurons within the telencephalon of five different species, including zebra finches, ferrets, gerbils and marmosets, in addition to mice. Outside of developing one’s own virus, the best strategy for achieving expression in a new system may be to assemble a panel of viruses from multiple sources and to systematically test them side by side. Viral vector cores appear increasingly willing to distribute test panels containing small aliquots of samples. Together with donations from labs that create new virus options, simultaneous—rather than sequential—testing could speed the outcome for selecting the best option.

## Abbreviations

AAV: Adeno-associated virus
ChR2: Channel rhodopsin
CMV: Cytomegalovirus
CREB: Cyclic AMP response-element binding protein
DM: Dorsomedial nucleus of the intercollicular complex
DLM: Dorsolateral nucleus of the medial thalamus
FoxP2: Forkhead box P2 transcription factor
GFP: Green fluorescent protein
GPe: Globus pallidus pars externa
GPi: Globus pallidus pars interna
HSV: Herpes simplex virus
hSyn1: Human synapsin 1
HVC: Acronym used as a proper name
IRES: Internal ribosomal entry site
Lant6: Lys8-Asn9-neurotensin8-13
LMAN: Lateral magnocellular nucleus of the anterior nidopallium
MSN: Medium spiny neuron
nXIIts: Tracheosyringeal part of nucleus XII
PGK: Phosphoglycerate kinase
RA: Robust nucleus of the arcopallium
TSA: Tyramide signal amplification
YFP: Yellow fluorescent protein
